# Assessing the impact of a gender-neutral approach to HPV vaccination on vaccination coverage for nine-year-old girls in Cameroon: A retrospective, cross-sectional study

**DOI:** 10.1371/journal.pgph.0006349

**Published:** 2026-09-09

**Authors:** Bridget C. Griffith, Shariffatou Iliassu, Clarence Mbanga, Budzi Michael Ngenge, Sonali Patel, Justin C. Graves, Navpreet Singh, Shalom Ndoula, Andreas Ateke Njoh, Efouba Gisele, Shadrack Mngemane, Tosin Ajayi, Laure-Anais Zultak, Yauba Saidu

**Affiliations:** 1 Analytics and Implementation Research Team, Clinton Health Access Initiative, Boston, Massachusetts, United States of America; 2 Clinton Health Access Initiative, Tsinga Sous-Prefecture, Yaoundé, Cameroon; 3 Global Vaccines Delivery Team, Clinton Health Access Initiative, Boston, Massachusetts, United States of America; 4 Expanded Program on Immunization, Messa, Yaoundé, Cameroon; 5 School of Global Health and Bioethics, Euclid University, Bangui, Central African Republic; 6 Institute for Global Health, University of Siena, Siena, Italy; 7 Department of Environmental and Global Health, College of Public Health & Health Professions, University of Florida, Gainesville, Florida, United States of America; University of Cape Town, SOUTH AFRICA

## Abstract

Cameroon introduced the human papilloma virus vaccine (HPVV) into the routine immunization schedule in October 2020. By the end of 2022, coverage remained low. To increase coverage, Cameroon switched to a country-wide, gender-neutral vaccination (GNV) approach in 2023, coupled with a revamped delivery strategy of Community Dialogues (CDs) and Periodic Intensification of Routine Immunization (PIRIs) activities in selected health districts (HDs). We assessed the impact of these programmatic changes, notably the GNV approach, on HPVV coverage. This retrospective, cross-sectional study measured the effect of GNV and CDs+PIRIs on HPVV coverage among 9-year-old girls in Cameroon (2022–2023). Data on HPV vaccination from all 203 HDs were extracted from DHIS2, and coveragecalculated at the HD level based on the estimated population eligible of 9-year-old girls. Descriptive statistics and multiple regression models were employed to assess the impact of GNV on vaccination coverage, while adjusting for CDs+PIRIs, baseline (2022) coverage, region, urban/rural status. In 2023, of the 203 HDs, 115 (56.7%) conducted GNV only and 74 (36.5%) implemented GNV & CDs+PIRIs. Among age-eligible girls, there was an overall increase in HPV vaccination coverage, with coverage rising 39.2 percentage points from 2022 to 2023. Following multiple linear regression, there was a significant association between percentage point increase in HPVV coverage in HDs with GNV & CDs+PIRIs, compared to those with no GNV and no CDs+PIRIs in the adjusted model (β:44.5, 95%CI: 29.4, 59.6, *p* < 0.001). Furthermore, there was a significant association between increase in HPVV coverage in HDs with GNV only compared to those with no GNV or no CDs+PIRIs in the adjusted model (β:15.5, 95%CI: 1.23, 29.8 *p* < 0.001). Overall, the GNV approach was associated with an increase in HPVV coverage for girls, with greater magnitude and predicted precision when implemented alongside CDs+PIRIs.

## Introduction

Human papillomavirus (HPV) infection is the most prevalent sexually transmitted infection worldwide, with approximately 80% of adults acquiring the virus during their lifetime [1,2]. The disease is caused by one or more of the nearly 200 existing HPV serotypes. At least 12 of these serotypes are known to be oncogenic, causing several types of cancers, including cervical, anal, oropharyngeal, vulvar, vaginal, and penile cancers [3,4]. The virus is responsible for nearly 99% of cervical cancer cases, which is the fourth most common cancer among women globally [3,5]. In 2022, over 660,000 new cervical cancer cases and 350,000 related deaths were recorded, with more than 90% of these deaths occurring in low- and middle-income countries (LMICs), particularly in sub-Saharan Africa [3].

The HPV vaccine (HPVV) prevents HPV infection and can lower the risk of developing HPV related cancers [6]. The HPVV first became available for females in the United States in 2006 [7]. As of December 2025, 156 countries and territories have the HPVV on their national routine immunization schedule [8–10]. Globally, HPVV coverage increased from 13% in 2019 to 21% in 2024 (first dose by age 15, females) and from 11% in 2019 to 18% in 2024 (last dose by age 15, females) [10,11].

Despite the availability of safe and effective HPVVs, coverage remains low in Sub-Saharan Africa, where the burden of HPV-related disease is highest [12]. This persistently low coverage has been attributed to multiple barriers to vaccine uptake, including supply chain limitations, misinformation, vaccine hesitancy, and sociocultural factors, which are particularly pronounced in LMICs and sub-Saharan Africa [13–16]. Innovative strategies are needed to overcome these barriers and expand vaccine coverage.

In Cameroon, cervical cancer ranks as the second most common cancer among women, with approximately 2,770 new cases and 1,770 deaths annually [17]. To combat this high burden, Cameroon introduced a two-dose schedule of the HPVV into the routine immunization program in October 2020, targeting nine-year-old girls. However, coverage remained critically low, with coverage for the first and second dose estimated at 20.3% and 5.3% respectively by December 2022, far below the nationally defined target coverage of 40% at the time of introduction. Published reports suggest that this low uptake was, in part, driven by insufficient stakeholder engagement, spillover misinformation from COVID-19 vaccination, multiple dose schedules, and misconceptions about the vaccine, including the fact that it was a tool designed to sterilize young girls [18,19].

To address these challenges with HPVV uptake, Cameroon’s National Immunization Technical Advisory Group (NITAG) recommended transitioning to a one-dose schedule in November 2022, following evidence from global studies demonstrating its comparable efficacy to a two-dose regimen [20]. Additionally, Cameroon’s NITAG advised implementing a gender-neutral vaccination (GNV) strategy to vaccinate boys alongside girls, making Cameroon the first LMIC to introduce GNV for HPVV. This change was based on the recognition of the potential for HPV transmission by men and the low female vaccination coverage [21,22]. The decision was backed by evidence from high-income settings, which suggest that vaccinating boys amplifies the public health impact of HPVV and addresses sociocultural barriers tied to girl-only vaccination strategies [23].

The GNV strategy and single-dose schedule were rolled out nationwide in January 2023, targeting nine-year-old boys and girls through health facilities, schools, and community sites. Prior to rollout, the EPI engaged religious groups, education authorities, civil society organizations, and community leaders at all levels of the health pyramid to counter vaccine misconceptions and build acceptance [22,24]. PIRI campaigns ran from March to June 2023, funded by Gavi, UNICEF, WHO, and the Government of Cameroon, extending vaccination beyond fixed facilities to households and schools to reach previously missed adolescents [22]. Community dialogues were implemented in selected health districts preceding or alongside PIRI activities. These dialogues involved structured community-level conversations, led by traditional and religious leaders, community health workers, and EPI focal points, aimed at dispelling myths, obtaining community and parental consent, and mobilizing target populations for vaccination [24]. In the northern regions, existing traditional community structures were leveraged to gather target age cohorts at the residences of traditional leaders or other community sites on pre-agreed vaccination days. Parent-teacher association meetings were also utilized to sensitize parents, obtain consent, and facilitate school-based vaccination [22]. Together, these complementary interventions integrated facility- and community-based approaches to address hesitancy and expand coverage across diverse settings.

Given the novelty of the GNV strategy in Cameroon and more broadly in LMICs, and the limited evidence on the effectiveness of GNV as well as on its coupling with community centred interventions such as CDS and PIRIs, there was a need to assess the effect of these approaches on improving HPVV coverage. To bridge this gap, this study aimed to describe the distribution of GNV, CDs, and PIRIs in Cameroon upon their rollout in 2023, assess the change in HPVV coverage among eligible girls pre- (2022) and post- (2023) the implementation of these approaches, and evaluate the impact of the approaches (GNV and GNV+ PIRIS+CDs) on the change in HPVV coverage. In doing so, this study provides insights into the implementation outcomes and effectiveness of these strategies, contributing to the evidence required to inform sustainable HPV vaccination policies in Cameroon and other LMICs.

## Methods

### Ethics statement

The study protocol was reviewed and approved by the Clinton Health Access Initiative’s internal Scientific and Ethical Review Committee (SERC). It was also approved by Cameroon’s National Ethics Committee for Human Health Research (CNERSH) (application number 2024/06/1686/CE/CNERSH/SP). Prior to protocol development, study PIs held validation meetings with national EPI to align on the study scope, study aims and intended data use. The data was accessed from the Cameroon District Health Information System (DHIS2) on the 30th of June 2024, and the authors had no access to any information that could identify individuals during or after the data collection.

### Study design, setting, and participants

We conducted a retrospective, cross-sectional analysis of national administrative HPVV coverage data in Cameroon to assess the effect of GNV, CDs, and PIRIs on HPVV coverage among age-eligible girls in Cameroon. Quantitative data, spanning a 24-month period (January 2022 to December 2023), were extracted from the DHIS2 to evaluate changes in HPVV coverage before and after the implementation of the GNV strategy in January 2023, alongside the use of CDs and PIRIs for HPV vaccination.

The study included data from all 203 HDs across the ten regions of Cameroon, given the nation-wide implementation scope of the interventions assessed (GNV, CDs, and PIRIS) in 2023. The 203 HDs were unevenly distributed across the ten regions, with the highest number of HDs located in the Far-North region of the country. Across the country, CDs and PIRIs were either organized by the national EPI or by HD leadership. The designation of a HD as urban and rural was based on the definition and designation provided by the Cameroon National Institute of Statistics [25].

### Definitions

Vaccination for HPV was defined as having received one dose of HPVV within the calendar year (HPV1).

The population of children who are eligible for HPV vaccination is based on the estimated number of 9-year-old girls and boys within each HD. Target populations (number of eligible girls, number of eligible boys) as calculated by the EPI, were estimated based on the proportion of 9-year-old girls and boys in the total population for 2022 and 2023, which was in turn estimated using data from Cameroon’s last census conducted in November 2005, adjusted for year over year population growth.

To confirm the presence of CDs and PIRIs in each HD, we conducted a review of all relevant documents (including NITAG reports, EPI monthly and annual reports) pertaining to GNV for HPVV-related CDs and PIRIs conducted. To confirm if some CDs and PIRIs were planned and financed sub-nationally, regional EPI coordinators were consulted to identify any HDs where HD-level HPV-related PIRI initiatives were conducted beyond the ones planned for and financed by the central EPI. In addition, we held calls with all EPI regional coordinators to check the implementation of sub-national (HD-level) CDs and PIRIs. The occurrence of CDs and PIRIs is referred to as “CDs+PIRIs” in the analysis.

The implementation of GNV for HPVV was initiated through a nationwide policy change, but in order to determine whether GNV occurred in a HD, the number of eligible boys vaccinated was collected. If zero eligible boys were vaccinated for HPV in 2023, the HD was categorized as having “no GNV” occur.

### Data collection, management, and validation

Data were extracted from the Cameroon DHIS2 immunization portal for all 203 HDs following approval from the EPI Permanent Secretary. The data consisted of two years (2022–2023) of anonymized administrative records, aggregated by Health Area, with no linkage to individual private information, ensuring confidentiality. The dataset included HPVV nationwide coverage for age-eligible girls (years 2022–2023) and boys (year 2023) by Health Area. To maintain data security, all files were stored on a password-protected server, accessible only to authorized personnel using individual logins and role-based permissions. Before commencing data extraction procedures, approvals were obtained from all relevant administrative bodies at national and sub-national levels. Upon completing the analysis, data files were deleted to ensure confidentiality and compliance with ethical standards.

We conducted data cleaning to identify and recode missing values, check for outliers and potential data entry errors, and address inconsistencies in formatting. Data cleaning, analysis, and visualization was conducted with Stata (Version 16) and Excel (Microsoft 365).

### Statistical analysis

HPVV coverage amongst eligible girls was calculated as the number of eligible girls vaccinated with one dose of the HPVV in each HD divided by the estimated number of 9-year-old girls in the HD. The same approach was used to calculate HPVV coverage amongst eligible boys.

Descriptive characteristics of HDs and interventions were summarized using frequencies and percentages. Vaccination totals and proportions were computed by summing the number of targeted and vaccinated girls annually, stratified by region, HD, and rural versus urban status. Due to variations in CDs+PIRIs implementation at the health area level, the unit of analysis was aggregated to the HD.

To assess the relationship between GNV, CDs+PIRIs and change in HPVV coverage among girls, we conducted two multiple linear regression models with change in HPVV coverage in girls from 2022 to 2023 as the continuous outcome. Multiple linear regression was selected over alternative models due to the continuous nature of the outcome variable and the ease of interpretation of model outputs. Beta regression was considered but ruled out due to the outcome variable (difference in HPVV coverage) values falling outside of the 0–1 range. Generalised linear models were considered but ruled out due to the normal distribution of residuals and constant variance assumptions being met.

Variables were selected for inclusion in the model *a priori,* and model diagnostics (residual plots, variance inflation factors, Q-Q plots of residuals, R^2^, and adjusted R^2^) was considered in selection of final models.

Model assumptions were assessed using standard diagnostic procedures and used to determine the inclusion or exclusion of variables. Residual plots (residuals vs. fitted values) for both models were examined to assess linearity and homoscedasticity, and no systematic patterns or funnelling were observed, suggesting that the assumptions of linearity and constant variable were reasonably met. Q-Q plots for both models indicated approximate normality of residuals. Variance inflation factors were below 5 for both models [(Model 1: range: 1.15, 4.66; mean: 2.0), (Model 2: range:.1.15, 2.14; mean: 1.5)], indicating no substantial multicollinearity among covariates. Both models explained about 50% of the variance in change in HPVV coverage (R^2^ = 0.50; adjusted R^2^ = 0.47), supporting the appropriateness of the fitted linear regression model. We present adjusted and unadjusted model outputs.

In the first model (Model 1), the dependent variable was the change in HPVV coverage in girls from 2022 to 2023. The independent variables were intervention (no GNV or CDs+PIRIs (ref.); GNV and CDs+PIRIs; GNV only), baseline (2022) HPVV coverage for girls (continuous), region (Centre(ref.); Adamawa; East; Far-North; Littoral; North; North-West; South; South-West; West), and urban/rural status(urban(ref.); rural).

In the second model (Model 2), the dependent variable was the change in HPVV coverage in girls from 2022 to 2023. The independent variables were GNV (no (ref.); yes), CDs+PIRIs (no (ref); yes), baseline (2022) HPVV coverage for girls (continuous), region (Centre(ref.); Adamawa; East; Far-North; Littoral; North; North-West; South; South-West; West), and urban/rural status(urban(ref.); rural).

Some HDs reported coverage values over 100%. We conducted a one-way sensitivity analysis to compare multiple methods to address these implausible values, including capping vaccine coverage values above 100%, setting vaccine coverage values over 100% to missing, and doing nothing to these values. This sensitivity analysis effected three (1.5%) of the 203 2022 HD coverage values and 52 (25.6%) of the 203 2023 HD coverage values. After comparing the model results and interpretation visually between the three scenarios, we decided to cap coverage values over 100% at 100%. The outputs of the models in which vaccine coverage values were capped at 100% are presented in this manuscript.

## Results

### HPV vaccination coverage in Cameroon 2022–2023

In 2022, of the 415,296 nine-year old girls targeted for vaccination with the HPVV, 80,168 (19.3%) were vaccinated with one dose of the vaccine. In 2023, 249,594 (58.5%) out of 426,429 girls targeted were vaccinated with one dose of the HPVV. In 2023, 112,606 (26.9%) of the 417,985 boys who were eligible for the HPVV were vaccinated (Table 1).

**Table 1:** Number and proportion vaccinated for HPVV.

Intervention (number of HDs)	Girls			Boys
	2022 Coverage n/N (%)	2023 Coverage n/N (%)	Coverage change (95% CI)	2023 coverage n/N (%)
**Overall (203)**	80,168 / 415,296 (19.3%)	249,594 / 426,429 (58.5%)	+39.2% (39.0, 39.4)	112,606 / 417,985 (26.9%)
**GNV only (115)**	32,445 / 238,654 (13.6%)	80,829 / 245,442 (32.9%)	+19.3% (19.1, 19.5)	34,988 / 240,581 (14.5%)
**GNV and CDs+PIRIs (74)**	47,219 / 166,953 (28.3%)	168,222 / 171,168 (98.5%)	+70.2% (70.0, 70.4)	77,618 / 167,778 (46.3%)
**No GNV or CDs+PIRIs (14)**	504 / 9,697 (5.2%)	143 / 9,820 (1.5%)	−3.7% (−4.2, −3.2)	0 / 9,625 (0%)

n = number vaccinated; N = target population.

In the 115 HDs where only GNV was implemented, the HPVV coverage increased from 13.6% in 2022 to 32.9% in 2023, a 19.3 percentage point increase. A larger increase in coverage from 2022 to 2023 was observed among the 74 HDs where GNV was implemented alongside CDs+PIRIs, with an increase in vaccination coverage from 28.3% in 2022 to 98.5% in 2023, a 70.2 percentage point increase (Table 1).

As presented in Table 2, among all HDs, the median proportion of eligible girls vaccinated for HPV in 2022 was 9.6% (IQR: 1.7, 31.0). This median proportion increased to 47.5% (IQR: 7.3, 100.0) in 2023. The greatest increase in coverage between 2022 and 2023 was observed amongst the HDs where both GNV and CDs+PIRIs occurred (54.0%; IQR: 24.9, 69.0). HDs that only implemented GNV also had an increase in coverage of 11.9% (IQR: 1.7, 38.2), while HDs that did not implement GNV or CDs+PIRIs had a minimal change in coverage (a decrease in some HDs) between the two years (0.27%; IQR: -1.3, 1.9).

**Table 2:** Median HPVV coverage for girls, per GNV intervention.

Intervention (number of HDs)	Overall (203)	GNV (115)	GNV& CDs+PIRIs (74)	No GNV or CDs+PIRIs (14)
**% vaccinated for HPVV 2022** **Median (IQR)**	9.6 (1.7 - 31.0)	4.9 (1.1 - 18.0)	25.6 (8.6 - 38.4)	0.88 (0 - 2.9)
**% vaccinated for 2023** **Median (IQR)**	47.5 (7.3 - 100.0)	24.3 (4.3 - 70.3)	100.0 (58.4 - 100.0)	1.0 (0.44 - 3.0)
**% change** **Median (IQR)**	22.0 (2.3 - 58.4)	11.9 (1.7 - 38.2)	54.0 (24.9 - 69.0)	0.27 (-1.3 - 1.9)

### Distribution of GNV and CDs+PIRIs

As presented in Table 3, out of the 203 HDs, about half (56.7%; n = 115) conducted GNV in 2023 without CDs+PIRIs. In about one third (36.5%; n = 74) of HDs, GNV was implemented alongside CDs+PIRIs, financed either at the national or HD-level. The remaining 14 HDs (6.9%) did not implement GNV (no eligible boys were vaccinated) or conduct CDs+PIRIs. Just over one fifth of the HDs (21.2%; n = 43) were classified as urban, while the rest were classified as rural (78.8%; n = 160).

**Table 3:** Description of health districts, by intervention.

Characteristic	Number of health districts (%)			
	Overall	GNV only	GNV and CDs+PIRIs	no GNV or CDs+PIRIs
**Overall**	203 (100)	115 (56.7)	74 (36.5)	14 (6.9)
**Region**				
Adamawa	11 (5.4)	4 (36.4)	7 (63.6)	0 (0.0)
Centre	32 (15.8)	20 (62.5)	7 (21.9)	5 (15.6)
East	15 (7.4)	8 (53.3)	7 (46.7)	0 (0.0)
Far-North	32 (15.8)	18 (56.3)	14 (43.8)	0 (0.0)
Littoral	24 (11.8)	13 (54.2)	7 (29.2)	4 (16.7)
North	15 (7.4)	7 (46.7)	8 (53.3)	0 (0.0)
North-West	21 (10.3)	16 (76.2)	4 (19.1)	1 (4.8)
South	12 (5.9)	7 (58.3)	5 (41.7)	0 (0.0)
South-West	21 (10.3)	13 (61.9)	8 (38.1)	0 (0.0)
West	20 (9.9)	9 (45.0)	7 (35.0)	4 (20.0)
**Median proportion (IQR)**		55.3 (15.8)	39.9 (21.0)	0.0 (15.9)
**Urbanicity**				
Rural	160 (78.8)	94 (58.8)	54 (33.8)	12 (7.5)
Urban	43 (21.2)	21 (48.8)	20 (46.5)	2 (4.7)

The proportion of HDs within a region that conducted each of the three implementation types (GNV only, GNV & CDs+PIRIs, and no GNV or CDs+PIRIs) varied across all regions, with a median of 55.3% (IQR = 15.8) of HDs per region involved in GNV only, 39.9% (IQR = 21.0) in GNV & CDs+PIRIs, and 0.0% (IQR = 15.9) in no GNV or CDs+PIRIs.

### Effect of GNV and CDs+PIRIs on HPVV coverage

**Fig 1:**
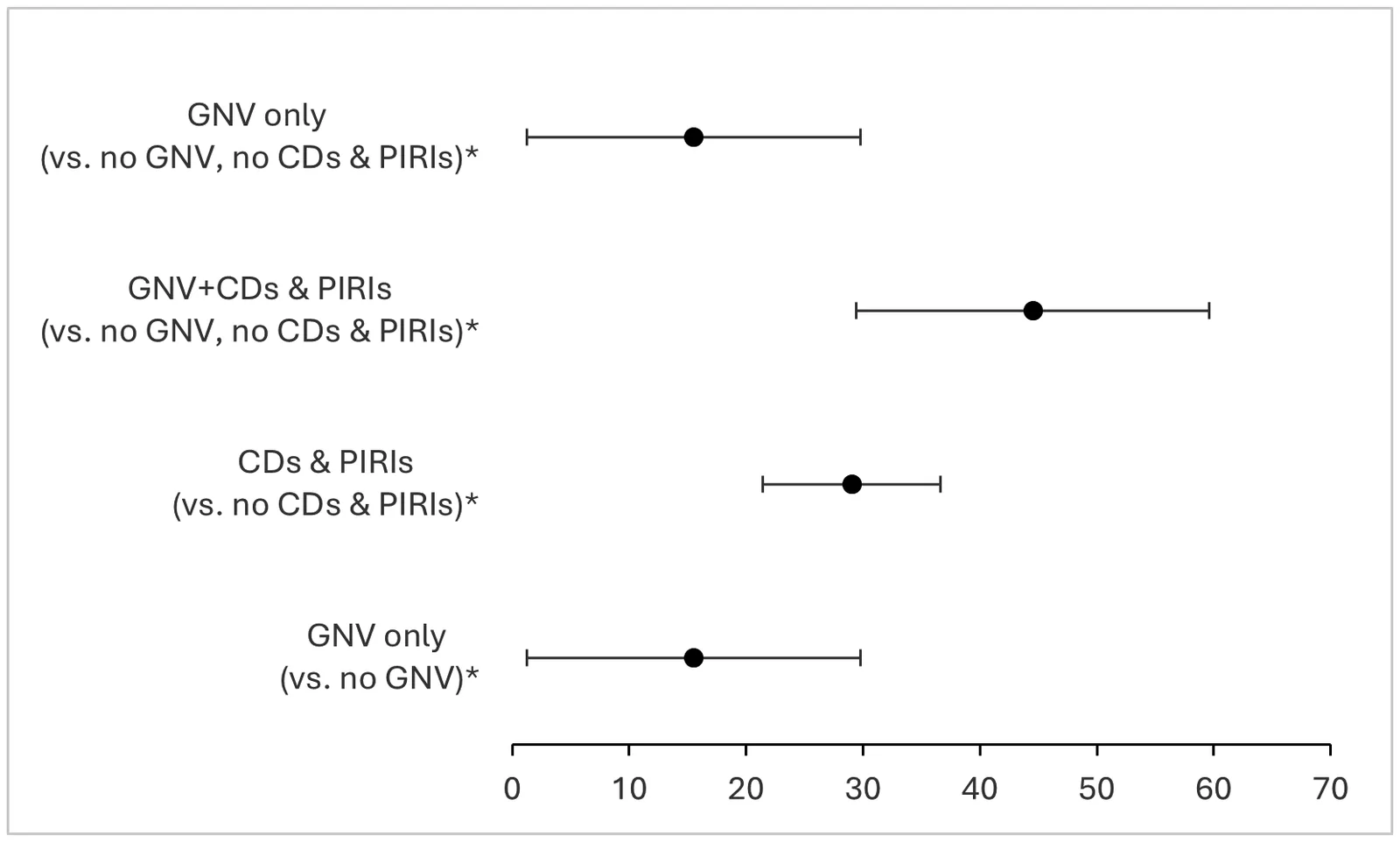
Adjusted model output (coefficients and 95% confidence intervals) from multiple linear regression models. Output from the models assessing the relationship between the listed independent variables and change in HPVV coverage among girls. *Indicates adjusted model output.

To measure the effect that GNV and GNV & CDs+PIRIs had on the change in HPVV coverage for girls, we conducted multiple linear regression models, with change in vaccine coverage from 2022 to 2023 as the continuous outcome (Fig 1; Tables 4, 5).

**Table 4:** Multiple linear regression of the combined effect of GNV & CDs+PIRIs on change in HPVV coverage output.

Dependent: Change in HPVV coverage					
	N (%)	Coef. (Unadjusted)	95% CI; p-value	Coef. (Adjusted)	95% CI; p-value
**Intervention**					
**No GNV or CDs+PIRIs (ref.)**	14 (6.9)	–	–	–	–
**GNV and CDs+PIRIs**	74 (36.5)	53.8	36.8, 70.8; *p* < 0.001	44.5	29.4, 59.6; *p* < 0.001
**GNV only**	115 (56.7)	28.2	11.7, 44.7; *p* = 0.001	15.5	1.2, 29.8; *p* = 0.03
**Baseline coverage**	–	–	–	-0.6	-0.8, -0.5
**Region**					
**Centre (ref.)**	32 (15.8)	–	–	–	–
**Adamawa**	11 (5.4)	–	–	40.9	23.4, 58.4; *p* < 0.001
**East**	15 (7.4)	–	–	38.0	22.6, 53.5; *p* < 0.001
**Far-North**	32 (15.8)	–	–	40.9	27.4, 54.4; *p* < 0.001
**Littoral**	24 (11.8)	–	–	-1.5	-14.5, 11.5; *p = 0.82*
**North**	15 (7.4)	–	–	39.7	23.0, 56.3; *p* < 0.001
**North-West**	21 (10.3)	–	–	14.0	-0.1, 28.1; *p = 0.05*
**West**	20 (9.9)	–	–	-5.1	-19.1, 8.8; *p = 0.47*
**South**	12 (5.9)	–	–	11.9	-4.7, 28.5; *p = 0.16*
**South-West**	21 (10.3)	–	–	21.7	7.5, 35.8; *p = 0.01*
**Urbanicity**					
**Rural (ref.)**	160 (78.8)	–	–	–	–
**Urban**	43 (21.2)	–	–	-9.8	-18.6, -0.9; *p* = 0.03

**Table 5:** Multiple linear regression of the individual effect of GNV and CDs+ PIRIs on change in HPVV coverage output.

Dependent: Change in HPVV coverage					
	N (%)	Coef. (Unadjusted)	95% CI; p-value	Coef. (Adjusted)	95% CI; p-value
**Presence of GNV**					
**No GNV (ref.)**	14 (6.9)	–	–	–	–
**GNV**	189 (93.1)	38.2	20.8, 55.6; *p* < 0.001	15.5	1.2, 29.8; *p* = 0.03
**Presence of CDs+PIRIS**					
**No CDs+PIRIs (ref.)**	129 (63.6)	–	–	–	–
**CDs+PIRIs**	74 (36.5)	28.6	19.9, 37.4; *p* < 0.001	29.0	21.4, 36.6; *p* < 0.001
**Baseline coverage**	–	–	–	-0.6	-0.8, -0.5; *p* < 0.001
**Region**					
**Centre (ref.)**	32 (15.8)	–	–	–	–
**Adamawa**	11 (5.4)	–	–	40.9	23.4, 58.4; *p* < 0.00
**East**	15 (7.4)	–	–	38.0	22.6, 53.5; *p* < 0.00
**Far-North**	32 (15.8)	–	–	40.9	27.4, 54.4; *p* < 0.00
**Littoral**	24 (11.8)	–	–	-1.5	-14.5, 11.5; *p = 0.82*
**North**	15 (7.4)	–	–	39.7	23.0, 56.3; *p* < 0.00
**North-West**	21 (10.3)	–	–	14.0	-0.1, 28.1; *p = 0.05*
**West**	20 (9.9)	–	–	-5.1	-19.1, 8.8; *p = 0.47*
**South**	12 (5.9)	–	–	11.9	-4.7, 28.5; *p = 0.16*
**South-West**	21 (10.3)	–	–	21.7	7.5, 35.8; *p = 0.01*
**Urbanicity**					
**Rural (ref.)**	160 (78.8)	–	–	–	–
**Urban**	43 (21.2)	–	–	-9.8	-18.6, -0.9; *p* = 0.03

Results from the adjusted multiple linear regression models are presented in a forest plot to visually compare model estimates and 95% confidence intervals (Fig 1). As shown in the figure, GNV and CDs+PIRIs was associated with the highest percentage point increase (44.5) in coverage when compared to no GNV or no CDs+PIRIs (95%CI: 29.4, 59.6). CDs+PIRIs were associated with a slightly lower unit change in coverage (29.0) but with greater precision due to the narrower 95% confidence interval (95%CI: 21.4, 36.6). When comparing GNV only to no GNV or CDs+PIRIS and to no GNV, we observed a lower percentage point increase in HPVV coverage (15.5 in both models) with a wider 95% confidence interval (95%CI: 1.23-, 29.8), but the adjusted effect of GNV on percentage point change in HPVV coverage remained directionally positive in both models.

In the model assessing the relationship between the presence of GNV and CDs+PIRIs and change in vaccine coverage, HDs with GNV and CDs+PIRIs were predicted to have a 44.5 percentage point increase in coverage (95%CI: 29.4, 59.6), compared to HDs with no GNV or CDs+PIRIs, while controlling for baseline coverage, region, and urban/rural status. Furthermore, HDs with GNV only were predicted to have a 15.5 percentage point increase in vaccine coverage (95%CI: 1.23, 29.8), when compared to HDs with no GNV or CDs+PIRIs (Table 4, Fig 1).

We also assessed the effect of CDs+PIRIs and GNV individually on the difference in HPVV coverage in 2022 and 2023. To do this, we ran an additional multiple linear regression model with change in vaccine coverage as the continuous outcome, and GNV (yes/no), CDs+PIRIs (yes/no) as independent variable, in addition to controlling for baseline coverage, region, and urban/rural status (urban/rural/NA). HDs with GNV were predicted to have a 15.5 percentage point increase in coverage (95%CI: 1.23-, 29.8), compared to HDs with no GNV. HDs with CDs+PIRIs were predicted to have a 29.0 percentage point increase in vaccine coverage (95%CI: 21.4, 36.6), when compared to districts with no CDs+PIRIs (Table 5, Fig 1).

### Sensitivity analysis

In the sensitivity analysis, we re-ran Models 1 and 2 with no change to the implausible coverage values, setting the implausible coverage values to missing, and capping the implausible coverage clause at 100%. In Table 6, we present the adjusted coefficients, 95% confidence intervals, and p-values for each scenario. The overall directionality of the associations did not change within the sensitivity analysis. Based on the outputs of the sensitivity analysis, capping coverage values > 100% at 100% was chosen, because it preserved the number of coverage values within the dataset and had a more marginal effect on the model output, compared to setting the coverage values > 100% to missing.

**Table 6:** Results from the three scenarios considered in the one-way sensitivity analysis.

		No change to coverage values >100%		Coverage values >100% set to missing		Coverage values >100% capped at 100%	
		N (%)	Coef. (Adjusted); 95% CI; p-value	N (%)	Coef. (Adjusted); 95% CI; p-value	N (%)	Coef. (Adjusted); 95% CI; p-value
**Model 1**	**No GNV or CDs+PIRIs (ref.)**	14 (6.9)	–	14 (6.9)	–	14 (6.9)	–
	**GNV and CDs+PIRIs**	74 (36.5)	67.5; 37.9, 97.0; *p* < 0.001	36 (17.7)	33.5; 19.5, 47.6; *p* < 0.001	74 (36.5)	44.5; 29.4, 59.6; *p* < 0.001
	**GNV only**	115 (56.7)	11.3; -16.7, 39.3; *p = 0.43*	100 (49.3)	11.6; -1.1, 24.2; *p = 0.07*	115 (56.7)	15.5; 1.2, 29.8; *p* = 0.03
							
**Model 2**	**No GNV (ref.)**	14 (6.9)	–	14 (6.9)	–	14 (6.9)	–
	**GNV**	189 (93.1)	11.3; -16.7, 39.3; *p = 0.43*	136 (67.0)	11.6; -1.1, 24.2; *p = 0.07*	189 (93.1)	15.5; 1.2, 29.8; *p* = 0.03
	**No CDs+PIRIs (ref.)**	129 (63.6)	–	114 (56.2)	–	129 (63.6)	–
	**CDs+PIRIs**	74 (36.5)	56.2; 41.3, 71.1; *p* < 0.001	36 (17.7)	22.0; 13.5, 30.4; *p* < 0.001	74 (36.5)	29.0; 21.4, 36.6; *p* < 0.001

## Discussion

We conducted a retrospective analysis of HPVV data from 2022 to 2023 to determine the effect that GNV and CDs+PIRIs had on HPVV coverage for eligible-aged girls in Cameroon. This study highlights the positive association between the Gender-Neutral Vaccination (GNV) approach and HPVV coverage, suggesting a substantial increase in coverage for girls in 2023.

The implementation of GNV and CDs+PIRIs varied considerably across HDs, likely reflecting the complexities of scaling a novel vaccination strategy nationwide. Just over half of HDs (56.7%) implemented GNV alone, while just over a third (36.5%) paired GNV with CDs+PIRIs, and a small minority (6.9%) implemented neither. This distribution suggests that the combined GNV and CDs+PIRIs approach was not the default implementation model in most districts, potentially reflecting logistical, financial, or capacity constraints associated with layering community-centred interventions onto the GNV platform. The considerable regional variation in implementation approach (proportion of HDs conducting GNV and CDs+PIRIs ranging from 19.1% in some regions to 63.6% in others) points to differences in sub-national readiness, resource allocation, or local prioritization which likely shaped the differential impact on HPVV coverage observed across regions. Notably, pairing of GNV with CDs+PIRIs was slightly more common in urban areas (46.5%) than rural areas (33.8%), which may reflect greater ease of coordinating and financing community-based interventions where infrastructure and health system capacity are more developed. While our model findings suggest that there is a negative association between urban HDs and change in coverage, this finding might have more to do with differences in implementation challenges between urban and rural areas and a lower baseline coverage in urban areas than a direct relationship between GNV, urban HDs, and coverage change. These implementation patterns underscore that GNV’s scale-up in Cameroon was uneven, and that the observed variation in coverage gains across districts may be attributable, in part, to this heterogeneity in the depth and combination of strategies deployed, rather than to differences in the effectiveness of GNV alone.

The substantial national improvement in HPVV coverage among girls pre- (19.3% in 2022) and post- (58.5% in 2023) GNV points to a meaningful role that the strategy played in closing the vaccination gap that had persisted prior to its rollout. Even where GNV was implemented alone, coverage more than doubled (13.6% to 32.9%), indicating that expanding vaccine availability through GNV was, in itself, an effective lever for improving uptake. This effect was considerably amplified, however, in districts that paired GNV with CDs+PIRIs, where 2022 coverage rose by about 70 percentage points to 98.5% in 2023. This suggest that community engagement builds substantially on the gains achieved by GNV alone, likely by addressing barriers such as awareness, trust, and access that expanding vaccine availability alone may not fully resolve. The contrast with districts that implemented neither intervention, where coverage stagnated and even declined slightly (5.2% to 1.5%), further underscores that the gains observed elsewhere were driven by active implementation efforts rather than by secular trends in vaccination behaviour. Taken together, these findings suggest that GNV represents an effective foundation for improving HPVV coverage, and that its integration with community-based mobilization further strengthens its impact, positioning the combination of GNV and CD+PIRIs as the more effective strategy for maximizing vaccination uptake at scale.

While GNV increased the proportion of girls vaccinated for HPV, the combination of GNV and CDs+PIRIs was significantly associated with a larger increase in the proportion vaccinated, based on the outputs of multiple linear regression models. This suggests that CDs+PIRIs play a key role in substantial coverage gains on top of the GNV policy and represent an important component of national and subnational vaccination efforts. Carrying out frequent CDs and PIRIs promotes interaction and engagement with community members and Civil Society Organizations (CSOs), allowing for the handling of issues and misconceptions about vaccination, and ultimately enhancing acceptance and uptake among families [26,27]. A previously published study on the GNV strategy for HPVV in Cameroon describes the HPVV implementation process, GNV rollout and CDs+PIRIs timeline in detail [22]. This study suggests that PIRIs are critical in significantly increasing the uptake of HPVV among both boys and girls in Cameroon, using a segmented regression with interrupted time series methodology. In addition, this study estimated HPVV coverage at the regional level, highlighting that the WHO recommend threshold for HPVV coverage of 90% was achieved in some regions as a result of the combined efforts of GNV, CDs, and PIRIs [5,22]. Our study builds on this work by describing the effect of GNV and CDs+PIRIs on change in HPVV coverage at a national level and demonstrating a method for managing out of range vaccine coverage estimates using sensitivity analysis.

Gender neutral HPV vaccination strategies are identified as a more inclusive approach to disease prevention, including evidence that vaccination of boys can reduce the transmission of HPV [28] and reduce the risk of HPV-related cancers in men [29]. This is also motivated by the estimate that 80% HPVV coverage is needed among girls to reduce HPV infection in boys, and current global and regional coverage remains lower than that threshold [6]. This inclusive approach has been highlighted as an effective strategy to comprehensively address HPV-driven disease and improve coverage of HPVV in sub-Saharan Africa [30], but there is minimal evidence for or against the expansion of HPV vaccination to include boys specifically to increase HPVV uptake among girls. This is among one the first studies to assess the impact of GNV on HPVV coverage among girls.

There remains a debate about the cost effectiveness of expanding HPV vaccination to males, in which cost effectiveness depends heavily on the vaccine coverage of females [31]. In addition, economic evaluations of the cost effectiveness in LMICs and sub-Saharan Africa are extremely limited. While this study does not directly address costs, it does demonstrate a potential practical benefit to vaccinating boys (an increase in uptake among girls). Future studies of real-world implementation on gender neutral HPVV should include a measure of the change in HPVV uptake among girls. In addition, an increase in the number of years of data will improve the precision of these findings.

The effective implementation of GNV and associated delivery strategies, such as PIRIs, however, require sustained financial and human resources. Evidence from an earlier qualitative assessment of the GNV approach in Cameroon demonstrated that resource constraints, including staff shortages, inadequate funding for transportation, personnel training, and community mobilization greatly hindered implementation at the health facility and community levels [24]. These operational resource demands were further compounded by the fact that expanding vaccination to include boys for the GNV approach required an additional USD 506,745 in vaccine costs alone, while implementing PIRIs necessitated multi-donor financing from international partners and the Government of Cameroon [22]. Future studies of GNV in Cameroon should include cost effectiveness analyses to fully understand the cost implications of this intervention. Critically, because Gavi predominantly directs vaccine procurement resources toward girls, countries adopting GNV must independently secure funding for male vaccination, creating a financing gap that threatens the sustainability of the approach [22]. These collectively underscore that securing sufficient and sustainable funding is not merely an operational consideration but a prerequisite for achieving the intended public health impact of GNV and CDs+PIRIs.

This study has several limitations. The observational design lacks randomization, which may have introduced selection bias and limited the ability to establish causal relationships between the GNV approach and vaccination outcomes. In addition, the lack of randomization meant the intervention areas were not balanced, and the places without CDs+PIRIs could possibly be correlated with low coverage or other coverage-influencing factors. The lack of visibility into these factors may cause unaccounted for biases in the analysis and interpretation. In this setting, CDs+PIRIs were always done in tandem, although they represent distinct implementation strategies, which is why they are grouped together throughout the analysis. Due to the combined nature of the reporting, we are unable to draw conclusions on the individual effects of these interventions on HPVV coverage.

Our study relied on retrospective administrative data from DHIS2 that was not collected for research; it contained some missing and inconsistent values, such as coverage estimates over 100%, and it could be a source of reporting biases and other inaccuracies. Coverage estimates over 100% may be due to the use of estimated population numbers based on 2005 census data as a denominator. In addition, dependence on the 2005 census data for population estimates may be more likely to introduce denominator inaccuracies in regions with population displacement or unique population growth patterns since 2005. We addressed the challenge of coverage estimates by changing the unit of analysis from health area to HD and conducting sensitivity analyses to ascertain the effect the nonsensical values had on model outputs. Reporting biases and inaccuracies in the data were addressed using data cleaning where possible. In addition, administrative data reporting the presence of CDs+PIRIs by geographical unit may lead to mis-categorization and bias, as the reality of the presence of these interventions may be different at the individual level. Similarly, the GNV exposure is defined as any vaccination of boys within the HD, as reported in the administrative data, which may not reflect intensity or coverage of implementation. Due to the limitations of the administrative retrospective data, we have to assume the GNV policy was implemented everywhere, and exposure is valid when we see any boys vaccinated. Lastly, because this design is observational in nature it is primarily suited for identifying potential associations and hypothesis generation, rather than causal inference.

## Conclusion

This analysis of administrative data demonstrates that the Gender-Neutral Vaccination (GNV) approach is associated with an increase in HPVV coverage in girls and therefore may be an effective strategy in increasing HPVV coverage in girls, particularly when accompanied with CDs and PIRIs. The implications of this study may be useful for other countries considering a GNV strategy to address low HPVV coverage, especially when low coverage is due to gender-related rumours. Future research should focus on measuring this relationship over longer periods of time and further differentiating between the effects that GNV, CDs, PIRIs, and a combined approach have on HPVV uptake, as well as the cost implications of these approaches. Overall, investment in research will be crucial for sustaining and expanding the positive impacts of the GNV approach, ensuring that no child is left unprotected against HPV-related cancers.

## Supporting information

S1 Data Full analysis dataset for all Health Districts. (CSV)
