# Supplementary material for: Assessing the impact of a gender-neutral approach to HPV vaccination on vaccination coverage for nine-year-old girls in Cameroon: A retrospective, cross-sectional study
Source: PLOS Glob Public Health. 2026 Sep 9;6(9):e0006349. doi: 10.1371/journal.pgph.0006349 (PMC13557348; doi:10.1371/journal.pgph.0006349)
Supplement: Supplementary file 1 [file pgph.0006349.s002.docx]

Summary of revisions:

Many thanks to the detailed and thoughtful comments and suggestions from the reviewers. We appreciate the time taken to complete the review, and we are confident that the paper is stronger now thanks to the reviewer’s comments.

In response to the reviewer’s comments, we have proofread the document and corrected grammar, spelling, and spacing inconsistencies. In the abstract, we have adjusted the language to not over emphasize causality of the study findings and we have updated the numbers to reflect the new model output. In the introduction, we have added more detail on the previously published study of this work and added more of a description of the intervention. In the methods section, we shifted the ethics statement to the beginning. We also provided clarity on how populations numbers were estimates, and we added a paragraph on the model diagnostics used in the analysis. Based on the feedback from the reviewers, we updated our models to include additional covariates, so the description of the models is updated in the methods. In the results section, we simplified the tables for clarity, and we updated the numbers within the table to reflect the updated model outputs. The Figure 1 is now correctly formatted in the results section and in the accompanying file. We also added a section describing the results of the sensitivity analysis. In the discussion section, we added an additional description of the other study aims, as requested by a reviewer. We also added additional points of consideration to the study limitations paragraphs. Throughout the discussion, the causal language was replaced with language describing the association between GNV and increase in HPVV coverage.

| Comment | Response |
| --- | --- |
| 1. The ethical approval number(s) listed in the manuscript and/or submission metadata does not match the approval number on the ethical approval document you provided. Please ensure that all approval numbers are correct. | Thank you for pointing this out. The ethical approval number in the manuscript has been updated to match the approval document.(Line 123) |
| 2. Please amend your detailed Financial Disclosure statement. This is published with the article. It must therefore be completed in full sentences and contain the exact wording you wish to be published. i. Please clarify all sources of financial support for your study. List the grants, grant numbers, and organizations that funded your study, including funding received from your institution. Please note that suppliers of material support, including research materials, should be recognized in the Acknowledgements section rather than in the Financial Disclosure.  ii. State the initials, alongside each funding source, of each author to receive each grant. For example: "This work was supported by the National Institutes of Health (####### to AM; ###### to CJ) and the National Science Foundation (###### to AM)." iii. State what role the funders took in the study. If the funders had no role in your study, please state: “The funders had no role in study design, data collection and analysis, decision to publish, or preparation of the manuscript.” iv. If any authors received a salary from any of your funders, please state which authors and which funders. | The financial disclosure statement has been updated to the correct format. |
| 3. In the online submission form, you indicated that [Insert text from online submission form here].  All PLOS journals now require all data underlying the findings described in their manuscript to be freely available to other researchers, either  1. In a public repository,  2. Within the manuscript itself, or  3. Uploaded as supplementary information.  This policy applies to all data except where public deposition would breach compliance with the protocol approved by your research ethics board. If your data cannot be made publicly available for ethical or legal reasons (e.g., public availability would compromise patient privacy), please explain your reasons by return email and your exemption request will be escalated to the editor for approval. Your exemption request will be handled independently and will not hold up the peer review process, but will need to be resolved should your manuscript be accepted for publication. One of the Editorial team will then be in touch if there are any issues. | Thank you for pointing this out. The data will be submitted as part of the supplementary material. |
| 4. Please ensure that your Ethics Statement is available in its entirety at the beginning of your Methods section, under a subheading 'Ethics Statement'. | The “ethical considerations” section has been renamed to “ethics statement” and moved to the beginning of the methods section. (Lines 120-127) |
| 5. Please upload separate figure files in .tif or .eps format. Also, remove the figures from your manuscript file but keep the legends. For more information about figure files please see our guidelines:   https://journals.plos.org/globalpublichealth/s/figures  https://journals.plos.org/globalpublichealth/s/figures#loc-file-requirements  If the reviewer comments include a recommendation to cite specific previously published works, please review and evaluate these publications to determine whether they are relevant and should be cited. There is no requirement to cite these works unless the editor has indicated otherwise. | The figure file is now saved int he correct format, and the figure caption is contained within the manuscript file. |

Reviewer Comments

Reviewer #1:

Griffith et al., present a study on the impact of GNV coupled with CD and PIRIs on HPV vaccination coverage in Cameroon. The study assessed HPV coverage before and after the implementation of these interventions and reported a significant increase in HPV vaccination coverage for eligible girls.

This assessment is particularly relevant because HPV vaccination coverage in Cameroon remains low due to several factors, including misconceptions about the vaccine, such as fears that it may cause sterility. Therefore, strategies like GNV, which have been shown to improve HPV vaccine uptake, are important for increasing vaccination coverage and addressing barriers to vaccination.

| Comments | Response |
| --- | --- |
| Introduction |  |
| Generally, well written. | Thank you for this feedback |
| Line 91-97: The authors should explain how each intervention was carried out so readers may have a better understanding of the impact of each intervention | Thank you for this feedback. We have added more detail on each intervention into the introduction. (Lines 92-107) |
| Methods |  |
| Line 118: Correct to the Far North region | Thank you for pointing that out. The language is updated in the new version. (Line 137) |
| Results |  |
| The authors mention sensitivity analysis in the methods and discussion but no results have been provided on this analysis | Thank you for your comment. We have added a paragraph to the results section describing the results of the sensitivity analysis. (Lines 285-293) |
| Discussion |  |
| In the introduction, the authors mention 3 main objectives for their study but the discussion only touches on the impact of GNV, CD and PIRIs. Discussion points on the distribution of these interventions as well as the coverages before and after these interventions should be included in this section. | Thank you for pointing this out. We agree that this information was missing from the analysis. In the updated submission, we have added dedicated paragraphs to the discussion where we describe and discuss the findings of the two other objectives. (Lines 300-330) |
| Line 245-268: The authors should highlight the role PIRIs as a vaccination strategy play in improving vaccination coverage in routine immunisation as a whole. Then they can go on to describe how these PIRIs in association with GNV further improved coverage in their study. | Thank you for this feedback. The role of PIRIs and CDs is described in the discussion. In addition, the reasons for why PIRIs and CDs cannot be discussed individually is described in the limitations section of the discussion.(Lines 333-344) |
| Line 255: The authors mention that this analysis demonstrates that securing sufficient funding and resources is a critical investment for effective implementation…but nowhere in the introduction or results have they mentioned a relationship between funding and GNV or PIRIs. | Thank you for this feedback. We have removed that specific mention of funding in the manuscript, as we agree that it is off topic for the aims and scope of the analysis. |
| Line 263: The authors mention that their work builds on a study on the GNV strategy which was carried out in Cameroon. This study should be presented in the introduction as serve as part of the rationale for this current study. | Thank you for pointing this out. We added evidence from the previous study on GNV strategy to the introduction to provide context and 8rationale for our study. (Lines 86-98) |

Reviewer #2:

This manuscript presents an important and policy-relevant evaluation of the impact of a gender-neutral HPV vaccination (GNV) strategy on vaccination coverage among nine-year-old girls in Cameroon. The study addresses a critical gap in the literature, particularly in low- and middle-income countries (LMICs), where evidence on gender-neutral vaccination approaches remains limited. The use of national DHIS2 data across all 203 health districts strengthens the generalizability of the findings.

Major Strengths

The study leverages national-level administrative data, enhancing external validity. It addresses a novel and under-researched policy intervention (gender-neutral HPV vaccination in LMICs). The inclusion of complementary interventions (CDs + PIRIs) provides valuable programmatic insights. The use of multiple regression models and sensitivity analyses demonstrates an effort analytical rigor.

| Reviewer 1 Comments | Response |
| --- | --- |
| Major Concerns |  |
| 1. Study Design and Causality |  |
| The retrospective cross-sectional design limits causal inference. While the authors appropriately acknowledge this limitation, the manuscript occasionally uses language implying causality (e.g., “GNV increased coverage”), which should be tempered. The observed associations may be confounded by unmeasured factors such as baseline district performance, health system strength, or concurrent interventions. | Thank you for this feedback. We have tempered our use of language implying causality, and instead discuss the association between GNV and the increase in HPVV coverage. (Line 332) |
| 2. Potential Selection Bias and Confounding |  |
| There is a high likelihood of selection bias, as districts implementing CDs + PIRIs may systematically differ from those that did not. The manuscript notes this but does not sufficiently explore or adjust for potential confounders (e.g., baseline coverage, resource allocation, regional disparities). | Thank you for pointing this out. Based on your feedback, we considered adding multiple additional covariates to the model and settled on adding region and baseline (2022) coverage, based on the contextual relevance and the improvements in model fit. (Lines 201-209) |
| 3. Statistical Analysis Concerns |  |
| The use of linear regression on percentage change outcomes may be problematic, particularly given: The presence of coverage values exceeding 100% (as shown in Table 2, page 14). | Thank you for your feedback. We have updated the numbers displayed in Table 2 to match the dataset used in the regression analyses in which all coverage values are capped at 100%. (Lines 230-237) |
| Potential non-normality and heteroscedasticity of residuals. | Thank you for pointing this out. We added a description of how we addressed this into the model diagnostics paragraph in the methods. (Lines 191-200) |
| While sensitivity analyses were conducted, the rationale for selecting the “capped at 100%” model as primary should be more clearly justified. | Thank you for your comment. We have added a more detailed description of the sensitivity analysis in the Methods (lines 210-217) and results (Lines 286-293) |
| No discussion of model diagnostics (e.g., residual plots, multicollinearity) is provided. Consideration of alternative models (e.g., beta regression or generalized linear models) would strengthen robustness. | Thank you for your comment. We have added in a paragraph on model diagnostics to the methods and a description of why we chose a linear regression model. (Lines 191-200) |
| 4. Data Quality and Measurement Issues |  |
| The reliance on administrative DHIS2 data introduces risks of reporting bias and inaccuracies. | Thank you for pointing this out. We have added a direct mention of this to the limitations section of the discussion. (Lines 386-388) |
| Coverage values exceeding 100% suggest denominator estimation issues (based on 2005 census projections), which could significantly affect validity. | Thank you for you comment. We have addressed the use of coverage values over 100% using data cleaning and sensitivity analysis (Lines 210-217), which is described in the methods. Although this data has clear limitations, we decided to use it for this analysis due to its relevance to the Cameroonian EPI and Ministry of Health and the direct application and relevance to decision-making for the EPI. |
| The classification of GNV based on “zero boys vaccinated” may misclassify districts with implementation challenges rather than true absence of GNV. | Thank you for pointing this out. Normally the entire country was to implement GNV, however we gave this operational definition in the study because those districts did not vaccinate any boy. Despite being officially declared as implementing GNV. We doubt it was a problem with funding or resources and therefore don't think we can confidently pin that on lack of intentionality. |
| 5. Data Availability |  |
| The Data Availability Statement indicates that data are available “upon request,” which does not comply with PLOS data sharing policy. The authors should deposit anonymized datasets (or at minimum, aggregated district-level data used in analysis) in a public repository or provide a clear justification for restrictions. | Thank you for your comment. In this resubmission, we will provide an anonymized dataset in the supplemental materials to satisfy this policy from the journal. |
| Reviewer 2 Comments |  |
| Minor Concerns |  |
| 1. Clarity and Language |  |
| The manuscript is generally well written but requires minor edits: | Thank you for this feedback. |
| Typographical errors (e.g., “Staes” instead of “States,” page 6). | Thank you for pointing this out. The typo is corrected in the new version. (Line 64) |
| Occasional grammatical inconsistencies and spacing issues. | Thank you for pointing this out. We conducted an additional proofread to catch these issues. |
| Some sentences in the Discussion are overly long and could be streamlined. | Thank you for your comment. We have revised the discussion section to improve the overall flow and sentence structure. |
| 2. Presentation of Results Tables (e.g., Table 1 and Table 2) could be simplified for clarity. The presence of coverage >100% (page 14) should be more explicitly discussed in the Results, not only in Methods. | Thank you for your feedback. We have simplified Tables 1 and 2 for clarity. In addition, we have added a more detailed discussion of the coverage values >100% in the Results.(Lines 285-293) |
| Figure 1 is referenced but not sufficiently interpreted in the text. | Thank you for pointing this out. We have added a paragraph describing the interpretation of Figure 1. (Lines 260-268) |
| 3. Interpretation The discussion could better distinguish between:  Effects of GNV alone  Effects of community engagement strategies (CDs + PIRIs) | Thank you for your comment. We have added details to the discussion to highlight the effect of GNV alone vs. the effects of CDs + PIRIs. (Lines 333-338) |
| The conclusion may slightly overstate the effectiveness of GNV without sufficient causal evidence. | Thank you for your comment. We agree with this and have softened the language to emphasize the strength of the association between GNV and change in coverage, rather than implying causality. (Lines 331-338) |
| Ethical Considerations |  |
| Ethical approval was appropriately obtained from relevant bodies (CHAI SERC and CNERSH), and the use of anonymized administrative data is appropriate. No major ethical concerns identified. | Thank you for this feedback. |
| Overall Recommendation This is a valuable and timely manuscript with strong public health relevance. However, major revisions are required to address methodological limitations, improve statistical rigor, and ensure compliance with PLOS data sharing policies. | Thank you for this feedback. |

Reviewer #3:

This manuscript addresses an important and policy-relevant question on HPV vaccination strategies in Cameroon and provides useful real-world data from national DHIS2 systems. The topic is highly relevant to global cervical cancer elimination efforts. The dataset and national coverage are strengths. However, several methodological and interpretative issues should be addressed to strengthen the validity and clarity of the findings.

| Reviewer 3 Comments | Response |
| --- | --- |
| Causal language (Results, Discussion, Conclusion): The manuscript uses causal terms such as “effect” and “impact,” which are not fully supported by the observational cross-sectional design. Please revise wording throughout to reflect association rather than causation. | Thank you for mentioning this. We have updated the language in the abstract, discussion, and conclusion to not overstate the effect of the GNV on the outcome. (Lines 331-338) |
| Baseline confounding (Methods/Regression): 2022 baseline coverage is not included in regression models. This is likely a key confounder influencing both intervention allocation and 2023 outcomes. Consider adjusting for baseline coverage or modelling 2023 coverage with 2022 as a covariate, or provide a clear justification for its exclusion. | Thank you for pointing this out. In order to address this, baseline (2022) HPVV coverage was added to the final models in order to adjust for this variation. (Lines 201-209) |
| Outcome specification (Methods/Statistical analysis): The use of “change in coverage” as the primary outcome is sensitive to baseline variation. Please justify this approach more explicitly or consider an alternative model specification (e.g., adjusting 2023 coverage for 2022 values). | Thank you for pointing this out. In order to address this, baseline (2022) HPVV coverage was added to the final models in order to adjust for this variation. (Lines 201-209) |
| Exposure definition (Methods): GNV exposure is defined as any vaccination of boys, which may be too broad and may not reflect intensity or coverage of implementation. Please clarify rationale and acknowledge this limitation; alternative definitions could be considered if data allow. | Thank you for pointing this out. We have made this limitation of the administrative, retrospective data more clear in the limitations section of the discussion. We know that the national policy was implemented country wide, but implementation rigor was dependent on local authorities. With retrospective data, we have to assume policy was implemented everywhere, but we can only consider exposure when we see any boys vaccinated. |
| Combined interventions (Results/Discussion): CDs and PIRIs are grouped together despite being distinct implementation strategies. The manuscript should more clearly acknowledge that their individual effects cannot be disentangled. | Thank you for pointing out the combined nature of the reporting of CDs and PIRIs. A direct note to this has been added to the limitations section of the discussion. (Lines 382-385) |
| Coverage >100% (Results/Methods): The occurrence of coverage values exceeding 100% suggests denominator estimation issues. Please report how frequently this occurred and provide a clearer explanation. Consider moving sensitivity analysis details to supplementary material. | Thank you for this comment. We have added a more explicit description of how often this occurred and have added the original dataset to the supplemental materials. (Lines 213-214) |
| Statistical reporting (Results): Replace “p = 0.000” with “p < 0.001” throughout. | Thank you for identifying this error. I have updated the way p-values are reported in the tables. (Table 4, Table 5) |
| Terminology consistency (Throughout): Standardise terminology (e.g., HPV vaccine vs HPVV) for clarity and consistency. | Thank you for pointing this out. The language is now updated throughout the document. |
| Methods transparency: Please provide additional detail on regression diagnostics and how missing data were handled. | Thank you for this comment. We have added an additional paragraph to the methods on the regression diagnostics that were done for both models. (Lines 191-200) |
| Overall, this is a strong and relevant study. With clarification of analytical choices and more careful interpretation of associations versus causation, the manuscript would make a valuable contribution to the literature | Thank you for this feedback. |
