# Supplementary material for: Assessing the impact of a gender-neutral approach to HPV vaccination on vaccination coverage for nine-year-old girls in Cameroon: A retrospective, cross-sectional study
Source: PLOS Glob Public Health. 2026 Sep 9;6(9):e0006349. doi: 10.1371/journal.pgph.0006349 (PMC13557348; doi:10.1371/journal.pgph.0006349)
Supplement: Peer review history

# Peer review: Decision Letter 0

**DOI:** 10.1371/journal.pgph.0006349.r001

**Authors:** Edina Amponsah-Dacosta

27 Apr 2026

PGPH-D-26-01077

Assessing the impact of a gender-neutral approach to HPV vaccination on vaccination coverage for nine-year-old girls in Cameroon: a retrospective, cross-sectional study

PLOS Global Public Health

Dear Dr. Mbanga,

Thank you for submitting your manuscript to PLOS Global Public Health. After careful consideration, we feel that it has merit but does not fully meet PLOS Global Public Health’s publication criteria as it currently stands. Therefore, we invite you to submit a revised version of the manuscript that addresses the points raised during the review process.

Please submit your revised manuscript by Jun 26 2026 11:59PM. If you will need more time than this to complete your revisions, please reply to this message or contact the journal office at globalpubhealth@plos.org. When you’re ready to submit your revision, log on to https://www.editorialmanager.com/pgph/ and select the 'Submissions Needing Revision' folder to locate your manuscript file.

Please include the following items when submitting your revised manuscript:

- A letter that responds to each point raised by the editor and reviewer(s). You should upload this letter as a separate file labeled 'Response to Reviewers'.
- A marked-up copy of your manuscript that highlights changes made to the original version. You should upload this as a separate file labeled 'Revised Manuscript with Track Changes'.
- An unmarked version of your revised paper without tracked changes. You should upload this as a separate file labeled 'Manuscript'.

Guidelines for resubmitting your figure files are available below the reviewer comments at the end of this letter.

As the corresponding author, your ORCID iD is verified in the submission system and will appear in the published article. PLOS supports the use of ORCID, and we encourage all coauthors to register for an ORCID iD and use it as well. Please encourage your coauthors to verify their ORCID iD within the submission system before final acceptance, as unverified ORCID iDs will not appear in the published article. *Only*  the individual author can complete the verification step; PLOS staff *cannot*  verify ORCID iDs on behalf of authors.

We look forward to receiving your revised manuscript.

Kind regards,

Edina Amponsah-Dacosta, Ph.D., MPH

Academic Editor

PLOS Global Public Health

**Journal Requirements:**

1. The ethical approval number(s) listed in the manuscript and/or submission metadata does not match the approval number on the ethical approval document you provided. Please ensure that all approval numbers are correct.

2. Please amend your detailed Financial Disclosure statement. This is published with the article. It must therefore be completed in full sentences and contain the exact wording you wish to be published.

i. Please clarify all sources of financial support for your study. List the grants, grant numbers, and organizations that funded your study, including funding received from your institution. Please note that suppliers of material support, including research materials, should be recognized in the Acknowledgements section rather than in the Financial Disclosure.

ii. State the initials, alongside each funding source, of each author to receive each grant. For example: "This work was supported by the National Institutes of Health (####### to AM; ###### to CJ) and the National Science Foundation (###### to AM)."

iii. State what role the funders took in the study. If the funders had no role in your study, please state: “The funders had no role in study design, data collection and analysis, decision to publish, or preparation of the manuscript.”

iv. If any authors received a salary from any of your funders, please state which authors and which funders.

3. In the online submission form, you indicated that [Insert text from online submission form here].

All PLOS journals now require all data underlying the findings described in their manuscript to be freely available to other researchers, either

1. In a public repository,

2. Within the manuscript itself, or

3. Uploaded as supplementary information.

This policy applies to all data except where public deposition would breach compliance with the protocol approved by your research ethics board. If your data cannot be made publicly available for ethical or legal reasons (e.g., public availability would compromise patient privacy), please explain your reasons by return email and your exemption request will be escalated to the editor for approval. Your exemption request will be handled independently and will not hold up the peer review process, but will need to be resolved should your manuscript be accepted for publication. One of the Editorial team will then be in touch if there are any issues.

4. Please ensure that your Ethics Statement is available in its entirety at the beginning of your Methods section, under a subheading 'Ethics Statement'.

5. Please upload separate figure files in .tif or .eps format. Also, remove the figures from your manuscript file but keep the legends.

For more information about figure files please see our guidelines:

https://journals.plos.org/globalpublichealth/s/figures

https://journals.plos.org/globalpublichealth/s/figures#loc-file-requirements

If the reviewer comments include a recommendation to cite specific previously published works, please review and evaluate these publications to determine whether they are relevant and should be cited. There is no requirement to cite these works unless the editor has indicated otherwise.

**Additional Editor Comments (if provided):**

[Note: HTML markup is below. Please do not edit.]

Reviewers' comments:

Reviewer's Responses to Questions

**Comments to the Author**

1. Does this manuscript meet PLOS Global Public Health’s publication criteria?

Reviewer #1: Partly

Reviewer #2: Partly

Reviewer #3: Partly

**********

2. Has the statistical analysis been performed appropriately and rigorously?

Reviewer #1: Yes

Reviewer #2: No

Reviewer #3: No

**********

3. Have the authors made all data underlying the findings in their manuscript fully available (please refer to the Data Availability Statement at the start of the manuscript PDF file)?

The PLOS Data policy

Reviewer #1: Yes

Reviewer #2: No

Reviewer #3: Yes

**********

4. Is the manuscript presented in an intelligible fashion and written in standard English?

Reviewer #1: Yes

Reviewer #2: Yes

Reviewer #3: Yes

**********

**Reviewer #1:**  Griffith et al., present a study on the impact of GNV coupled with CD and PIRIs on HPV vaccination coverage in Cameroon. The study assessed HPV coverage before and after the implementation of these interventions and reported a significant increase in HPV vaccination coverage for eligible girls.

This assessment is particularly relevant because HPV vaccination coverage in Cameroon remains low due to several factors, including misconceptions about the vaccine, such as fears that it may cause sterility. Therefore, strategies like GNV, which have been shown to improve HPV vaccine uptake, are important for increasing vaccination coverage and addressing barriers to vaccination.

Comments

Introduction

Generally, well written.

Line 91-97: The authors should explain how each intervention was carried out so readers may have a better understanding of the impact of each intervention

Methods

Line 118: Correct to the Far North region

Results

The authors mention sensitivity analysis in the methods and discussion but no results have been provided on this analysis

Discussion

In the introduction, the authors mention 3 main objectives for their study but the discussion only touches on the impact of GNV, CD and PIRIs. Discussion points on the distribution of these interventions as well as the coverages before and after these interventions should be included in this section.

Line 245-268: The authors should highlight the role PIRIs as a vaccination strategy play in improving vaccination coverage in routine immunisation as a whole. Then they can go on to describe how these PIRIs in association with GNV further improved coverage in their study.

Line 255: The authors mention that this analysis demonstrates that securing sufficient funding and resources is a critical investment for effective implementation…but nowhere in the introduction or results have they mentioned a relationship between funding and GNV or PIRIs.

Line 263: The authors mention that their work builds on a study on the GNV strategy which was carried out in Cameroon. This study should be presented in the introduction as serve as part of the rationale for this current study.

**Reviewer #2:**  This manuscript presents an important and policy-relevant evaluation of the impact of a gender-neutral HPV vaccination (GNV) strategy on vaccination coverage among nine-year-old girls in Cameroon. The study addresses a critical gap in the literature, particularly in low- and middle-income countries (LMICs), where evidence on gender-neutral vaccination approaches remains limited. The use of national DHIS2 data across all 203 health districts strengthens the generalizability of the findings.

Major Strengths

The study leverages national-level administrative data, enhancing external validity.

It addresses a novel and under-researched policy intervention (gender-neutral HPV vaccination in LMICs).

The inclusion of complementary interventions (CDs + PIRIs) provides valuable programmatic insights.

The use of multiple regression models and sensitivity analyses demonstrates an effort toward analytical rigor.

Major Concerns

1. Study Design and Causality

The retrospective cross-sectional design limits causal inference. While the authors appropriately acknowledge this limitation, the manuscript occasionally uses language implying causality (e.g., “GNV increased coverage”), which should be tempered. The observed associations may be confounded by unmeasured factors such as baseline district performance, health system strength, or concurrent interventions.

2. Potential Selection Bias and Confounding

There is a high likelihood of selection bias, as districts implementing CDs + PIRIs may systematically differ from those that did not. The manuscript notes this but does not sufficiently explore or adjust for potential confounders (e.g., baseline coverage, resource allocation, regional disparities).

3. Statistical Analysis Concerns

The use of linear regression on percentage change outcomes may be problematic, particularly given:

The presence of coverage values exceeding 100% (as shown in Table 2, page 14).

Potential non-normality and heteroscedasticity of residuals.

While sensitivity analyses were conducted, the rationale for selecting the “capped at 100%” model as primary should be more clearly justified.

No discussion of model diagnostics (e.g., residual plots, multicollinearity) is provided.

Consideration of alternative models (e.g., beta regression or generalized linear models) would strengthen robustness.

4. Data Quality and Measurement Issues

The reliance on administrative DHIS2 data introduces risks of reporting bias and inaccuracies.

Coverage values exceeding 100% suggest denominator estimation issues (based on 2005 census projections), which could significantly affect validity.

The classification of GNV based on “zero boys vaccinated” may misclassify districts with implementation challenges rather than true absence of GNV.

5. Data Availability

The Data Availability Statement indicates that data are available “upon request,” which does not comply with PLOS data sharing policy. The authors should deposit anonymized datasets (or at minimum, aggregated district-level data used in analysis) in a public repository or provide a clear justification for restrictions.

Minor Concerns

1. Clarity and Language

The manuscript is generally well written but requires minor edits:

Typographical errors (e.g., “Staes” instead of “States,” page 6).

Occasional grammatical inconsistencies and spacing issues.

Some sentences in the Discussion are overly long and could be streamlined.

2. Presentation of Results

Tables (e.g., Table 1 and Table 2) could be simplified for clarity.

The presence of coverage >100% (page 14) should be more explicitly discussed in the Results, not only in Methods.

Figure 1 is referenced but not sufficiently interpreted in the text.

3. Interpretation

The discussion could better distinguish between:

Effects of GNV alone

Effects of community engagement strategies (CDs + PIRIs)

The conclusion may slightly overstate the effectiveness of GNV without sufficient causal evidence.

Ethical Considerations

Ethical approval was appropriately obtained from relevant bodies (CHAI SERC and CNERSH), and the use of anonymized administrative data is appropriate. No major ethical concerns identified.

Overall Recommendation

This is a valuable and timely manuscript with strong public health relevance. However, major revisions are required to address methodological limitations, improve statistical rigor, and ensure compliance with PLOS data sharing policies.

**Reviewer #3:**  This manuscript addresses an important and policy-relevant question on HPV vaccination strategies in Cameroon and provides useful real-world data from national DHIS2 systems. The topic is highly relevant to global cervical cancer elimination efforts. The dataset and national coverage are strengths. However, several methodological and interpretative issues should be addressed to strengthen the validity and clarity of the findings.

Causal language (Results, Discussion, Conclusion): The manuscript uses causal terms such as “effect” and “impact,” which are not fully supported by the observational cross-sectional design. Please revise wording throughout to reflect association rather than causation.

Baseline confounding (Methods/Regression): 2022 baseline coverage is not included in regression models. This is likely a key confounder influencing both intervention allocation and 2023 outcomes. Consider adjusting for baseline coverage or modelling 2023 coverage with 2022 as a covariate, or provide a clear justification for its exclusion.

Outcome specification (Methods/Statistical analysis): The use of “change in coverage” as the primary outcome is sensitive to baseline variation. Please justify this approach more explicitly or consider an alternative model specification (e.g., adjusting 2023 coverage for 2022 values).

Exposure definition (Methods): GNV exposure is defined as any vaccination of boys, which may be too broad and may not reflect intensity or coverage of implementation. Please clarify rationale and acknowledge this limitation; alternative definitions could be considered if data allow.

Combined interventions (Results/Discussion): CDs and PIRIs are grouped together despite being distinct implementation strategies. The manuscript should more clearly acknowledge that their individual effects cannot be disentangled.

Coverage >100% (Results/Methods): The occurrence of coverage values exceeding 100% suggests denominator estimation issues. Please report how frequently this occurred and provide a clearer explanation. Consider moving sensitivity analysis details to supplementary material.

Statistical reporting (Results): Replace “p = 0.000” with “p < 0.001” throughout.

Terminology consistency (Throughout): Standardise terminology (e.g., HPV vaccine vs HPVV) for clarity and consistency.

Methods transparency: Please provide additional detail on regression diagnostics and how missing data were handled.

Overall, this is a strong and relevant study. With clarification of analytical choices and more careful interpretation of associations versus causation, the manuscript would make a valuable contribution to the literature.

**********

what does this mean?). If published, this will include your full peer review and any attached files.

**Do you want your identity to be public for this peer review?**  If you choose “no”, your identity will remain anonymous but your review may still be made public.

For information about this choice, including consent withdrawal, please see our Privacy Policy

Reviewer #1: No

Reviewer #2: **Yes:**  Dr Alltalents T Murahwa

Reviewer #3: **Yes:**  Varsetile Varster Nkwinika

**********

[NOTE: If reviewer comments were submitted as an attachment file, they will be attached to this email and accessible via the submission site. Please log into your account, locate the manuscript record, and check for the action link "View Attachments". If this link does not appear, there are no attachment files.]

**Figure Resubmissions:**

# Author comment: Author response to Decision Letter 1

**DOI:** 10.1371/journal.pgph.0006349.r002

29 Jul 2026

Attachment Submitted filename: plos_sub1_responsetoreviewers_final.docx

# Peer review: Decision Letter 1

**DOI:** 10.1371/journal.pgph.0006349.r003

**Authors:** Edina Amponsah-Dacosta

6 Aug 2026

Assessing the impact of a gender-neutral approach to HPV vaccination on vaccination coverage for nine-year-old girls in Cameroon: a retrospective, cross-sectional study

PGPH-D-26-01077R1

Dear Dr Mbanga,

We are pleased to inform you that your manuscript 'Assessing the impact of a gender-neutral approach to HPV vaccination on vaccination coverage for nine-year-old girls in Cameroon: a retrospective, cross-sectional study' has been provisionally accepted for publication in PLOS Global Public Health.

Before your manuscript can be formally accepted you will need to complete some formatting changes, which you will receive in a follow up email. A member of our team will be in touch with a set of requests.

Please note that your manuscript will not be scheduled for publication until you have made the required changes, so a swift response is appreciated.

IMPORTANT: The editorial review process is now complete. PLOS will only permit corrections to spelling, formatting or significant scientific errors from this point onwards. Requests for major changes, or any which affect the scientific understanding of your work, will cause delays to the publication date of your manuscript.

If your institution or institutions have a press office, please notify them about your upcoming paper to help maximize its impact. If they'll be preparing press materials, please inform our press team as soon as possible -- no later than 48 hours after receiving the formal acceptance. Your manuscript will remain under strict press embargo until 2 pm Eastern Time on the date of publication. For more information, please contact globalpubhealth@plos.org.

Thank you again for supporting Open Access publishing; we are looking forward to publishing your work in PLOS Global Public Health.

Best regards,

Edina Amponsah-Dacosta, Ph.D., MPH

Academic Editor

PLOS Global Public Health

***********************************************************

Reviewer Comments (if any, and for reference):

Reviewer's Responses to Questions

**Comments to the Author**

Reviewer #1: All comments have been addressed

Reviewer #2: All comments have been addressed

Reviewer #3: All comments have been addressed

**********

publication criteria?

Reviewer #1: (No Response)

Reviewer #2: (No Response)

Reviewer #3: (No Response)

**********

3. Has the statistical analysis been performed appropriately and rigorously?

Reviewer #1: (No Response)

Reviewer #2: Yes

Reviewer #3: (No Response)

**********

4. Have the authors made all data underlying the findings in their manuscript fully available (please refer to the Data Availability Statement at the start of the manuscript PDF file)?

The PLOS Data policy

Reviewer #1: (No Response)

Reviewer #2: Yes

Reviewer #3: (No Response)

**********

5. Is the manuscript presented in an intelligible fashion and written in standard English?

Reviewer #1: (No Response)

Reviewer #2: Yes

Reviewer #3: (No Response)

**********

Reviewer #1: (No Response)

Reviewer #2: While the manuscript is strong, I offer the following suggestions for minor improvements:

Line 325-327: The text states "β:44.5%, 95%CI: 29.4, 59.6, p<0.001" but Table 4 shows "44.5" (without the percentage sign for the coefficient). For consistency, either use percentages throughout or clarify that the coefficient represents percentage points.

Interpretation of urban/rural findings: Table 4 shows a negative association between urban status and coverage change (β: -9.8, p=0.03). This is interesting and could be briefly discussed—perhaps urban areas had higher baseline coverage, leaving less room for improvement, or implementation challenges differ between settings.

Cost implications: The authors mention the additional cost of GNV (USD 506,745) but do not provide a cost-effectiveness analysis. While this is beyond the study's scope, a brief note on the need for future cost-effectiveness studies would be valuable.

Data limitations: Consider mentioning that using 2005 census data for population estimates may introduce denominator inaccuracies, particularly in regions with population displacement or growth patterns.

Reviewer #3: (No Response)

**********

what does this mean?). If published, this will include your full peer review and any attached files.

**Do you want your identity to be public for this peer review?**  If you choose “no”, your identity will remain anonymous but your review may still be made public.

For information about this choice, including consent withdrawal, please see our Privacy Policy

Reviewer #1: No

Reviewer #2: **Yes:**  Dr Alltalents T Murahwa

Reviewer #3: **Yes:**  Dr Varsetile Nkwinika

**********
